# Longitudinal morphological changes during recovery from brain deformation due to idiopathic normal pressure hydrocephalus after ventriculoperitoneal shunt surgery

**DOI:** 10.1038/s41598-019-53888-7

**Published:** 2019-11-21

**Authors:** Shigeki Yamada, Masatsune Ishikawa, Makoto Yamaguchi, Kazuo Yamamoto

**Affiliations:** 1 Department of Neurosurgery, Shiga University of Medical Science, Shiga, Japan; 2 Department of Neurosurgery and Normal Pressure Hydrocephalus Center, Rakuwakai Otowa Hospital, Kyoto, Japan; 3 Interfaculty Initiative in Information Studies/Institute of Industrial Science, The University of Tokyo, Tokyo, Japan; 4 Rakuwa Villa Ilios, Kyoto, Japan

**Keywords:** Hydrocephalus, Hydrocephalus

## Abstract

The present study aimed to examine time-dependent change in cerebrospinal fluid distribution and various radiological indices for evaluating shunt effectiveness in patients with idiopathic normal pressure hydrocephalus (iNPH). This study included 54 patients with iNPH who underwent MRI before and after ventriculoperitoneal shunt surgery. The volume of the total ventricles and subarachnoid spaces decreased within 1 month after shunting. However, more than 1 year after shunting, the volume of the total ventricles decreased, whereas that of the total subarachnoid spaces increased. Although cerebrospinal fluid distribution changed considerably throughout the follow-up period, the brain parenchyma expanded only 2% from the baseline brain volume within 1 month after shunting and remained unchanged thereafter. The volume of the convexity subarachnoid space markedly increased. The changing rate of convexity subarachnoid space per ventricle ratio (CVR) was greater than that of any two-dimensional index. The brain per ventricle ratio (BVR), callosal angle and z-Evans index continued gradually changing, whereas Evans index did not change throughout the follow-up period. Both decreased ventricular volume and increased convexity subarachnoid space volume were important for evaluating shunt effectiveness. Therefore, we recommend CVR and BVR as useful indices for the diagnosis and evaluation of treatment response in patients with iNPH.

## Introduction

Normal pressure hydrocephalus (NPH) was first proposed by Hakim and Adams as a surgically treatable cause of dementia in 1965^1^. With the development of imaging predictors of shunt effectiveness, the prevalence of idiopathic NPH (iNPH) has been increasing rapidly in developed countries characterized by a higher proportion of the elderly population^1–8^. Additionally, recent developed devices, such as adjustable valves with anti-siphon mechanisms, preoperative virtual simulators, and several intraoperative guiding tools, have improved the outcome of shunt surgery; approximately 70% of patients improve their symptoms to some extent^9–13^, and 10% of patients need shunt revision^10,11,14^. However, the symptoms of 30% of patients with iNPH who undergo shunt surgery are not improved for various reasons, including underdrainage, shunt malfunction, and comorbidities. If a patient’s symptoms do not improve or even worsen after shunt surgery, neurosurgeons must investigate the cause by computed tomography (CT) or magnetic resonance imaging (MRI). However, it is unknown how iNPH-specific indices change following shunt surgery.

**Figure 1:**
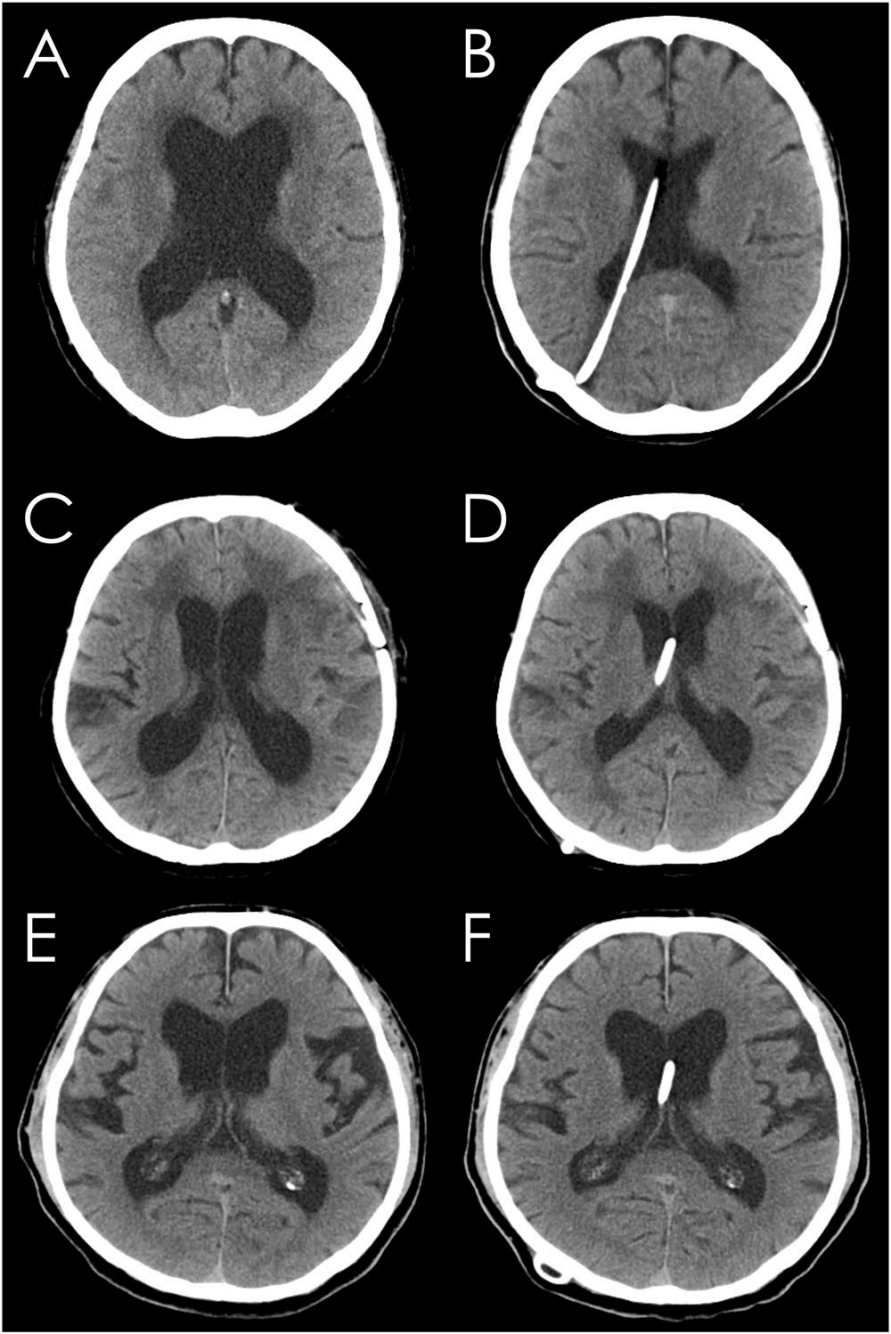
Conventional CT scan one day before and one month after shunt surgery. Representative cases diagnosed with adult-onset congenital NPH (**A**,**B**), secondary NPH (**C**,**D**) and idiopathic NPH (**E**,**F**).

According to Hakim’s hypothesis^15^, brain shrinkage due to compression from enlarged ventricles in NPH can be reverted by cerebrospinal fluid (CSF) volume subtraction. Indeed, in typical patients with secondary or congenital NPH, the brain parenchyma obviously expands as ventricular size decreases after CSF shunt surgery (Fig. 1A–D)^16,17^. However, the ventricular size measured by the Evans index remains often unchanged in elderly patients with iNPH (Fig. 1E,F), even if their symptoms are improved after shunting^18–21^. The key features of iNPH are compressed high-convexity sulci concurrent with enlarged Sylvian fissure and basal cistern, i.e., disproportionately enlarged subarachnoid space hydrocephalus (DESH)^4,22–24^. We recently showed that the pathophysiological mechanism of DESH in iNPH mainly involves the compensatory direct pathway of CSF between the inferior horn of the lateral ventricles and the basal cistern at the inferior choroidal point of the choroidal fissure^16^. Therefore, if the lateral ventricles and the basal cistern in iNPH are enlarged simultaneously, they may all become smaller following shunt surgery. Depending on the decrease in the volumes of the lateral ventricles and the basal cistern, the volume of the convexity subarachnoid space may increase. Therefore, we investigated the longitudinal change in iNPH-specific indices and the volumes in the brain parenchyma, ventricles, and subarachnoid spaces on three-dimensional (3D) MRI before and after shunt surgery. Additionally, because the presence of comorbid Alzheimer’s disease (AD) is associated with smaller, shorter-lasting effects of shunt treatment in patients with iNPH^25–27^, we also investigated whether the change in the indices and volumes is associated with clinical outcomes or comorbid AD.

## Results

### Clinical characteristics

In 54 consecutive patients with iNPH who underwent follow-up MRI within 1 month after ventriculo-peritoneal shunt (VPS) via parieto-occipital approach (mean age, 76.7 ± 5.8 years; range, 60 to 88 years; 35 men, 19 women), the longitudinal changes in the morphological indices for iNPH and the segmented volumes in brain parenchyma and CSF spaces were evaluated. The mean time interval from initial symptom onset until baseline MRI (before VPS) was 27.3 ± 19.7 months, and that from initial symptom onset until VPS was 48.7 ± 69.1 months. Follow-up MRI was conducted < 1 month (early) after VPS in all 54 patients, 1 month to 1 year (mid) in 28 patients, and > 1 year (late) in 16 patients. Table 1 presents the baseline clinical characteristics and outcome after VPS. The first 12 patients treated with a Codman Hakim programmable valve, whereas the latter 42 patients treated with a Codman CERTAS Plus programmable valve. There was no significant difference between the two groups in the mean values of age, disease duration, or frequency of co-morbidities and outcome. As shown in Table 2, the patients with pure iNPH had significantly larger Evans index and significantly smaller brain per ventricle ratios (BVRs) at the posterior commissure (PC) level at baseline MRI than those with co-occurrence of iNPH and AD. The other the iNPH-specific indices and segmented CSF volumes were not significantly different between patients with pure iNPH and those with co-occurrence of iNPH and AD.

**Table 1:** Clinical characteristics and outcome after shunt surgery.

Characteristics	Total (n = 54)	CHPV (n = 12) ~3. 2016	CERTUS (n = 42) 4. 2016~	*P* value*
Sex, men:women	35:19	9:3	26:16	0.542
Mean age ± SD, years	76.7 ± 5.8	75.2 ± 6.9	77.1 ± 5.3	0.220
Mean disease duration ± SD, years	2.3 ± 1.6	1.5 ± 1.1	2.5 ± 1.7	0.067
Mean days from MRI to shunt surgery	48.8 ± 69.1	39.7 ± 29.1	51.3 ± 76.9	0.906
Mean days from shunt surgery to the first follow-up MRI	12.2 ± 5.1	9.8 ± 2.6	12.9 ± 5.5	0.063
Comorbidity of Alzheimer’s disease	19	2	17	0.238
Outcome, excellent:good:unsatisfactory	43:11:0	8:4:0	35:7:0	0.217
Valve pressure at the first follow-up MRI		14.8 ± 3.9 mmH_2_O	4.5 ± 1.0	
Valve pressure at the second follow-up MRI		13.6 ± 3.7 mmH_2_O	4.1 ± 0.9	
Valve pressure at the third follow-up MRI		9.3 ± 2.8 mmH_2_O	3.4 ± 0.9	

Data are presented as the mean ± standard deviation unless otherwise noted.

^*^Probability value of the Wilcoxon rank-sum test: comparison between CHPV vs. CERTUS.

CERTUS, Codman CERTAS^®^ Plus programmable valve with Siphon-Guard^®^; CHPV, Codman Hakim programmable valve with Siphon-Guard^®^; MRI, magnetic resonance imaging; SD, standard deviation.

**Table 2:** Morphological indices, volumes, and VR at baseline MRI.

Characteristics	Total (n = 54)	Pure iNPH (n = 35)	iNPH + AD (n = 19)	*P* value*
Evans index (mean ± SD)	0.32 ± 0.06	0.34 ± 0.06	0.30 ± 0.05	**0**.**017**
z-Evans index (mean ± SD)	0.44 ± 0.06	0.45 ± 0.06	0.43 ± 0.06	0.173
Callosal angle (mean ± SD)	63.5 ± 18.5	61.6 ± 19.6	67.1 ± 16.2	0.193
BVR at the AC (mean ± SD)	0.72 ± 0.17	0.70 ± 0.17	0.77 ± 0.18	0.133
BVR at the PC (mean ± SD)	0.89 ± 0.23	0.82 ± 0.19	1.00 ± 0.24	**0**.**010**
CVR (mean ± SD)	0.47 ± 0.20	0.47 ± 0.21	0.48 ± 0.18	0.812
Total intracranial volume, mL	1543 ± 156	1562 ± 123	1509 ± 203	0.296
Brain parenchyma, mL (VR, %)	1107 (71.8)	1119 (71.7)	1084 (72.0)	0.388
Total CSF, mL (VR, %)	436.8 (28.2)	443.3 (28.3)	424.9 (28.0)	0.683
Total ventricle, mL (VR, %)	163.7 (10.5)	170.5 (10.9)	151.1 (9.9)	0.108
Bilateral ventricle, mL (VR, %)	155.0 (10.0)	161.5 (10.3)	143.0 (9.4)	0.126
Third ventricle, mL (VR, %)	5.2 (0.3)	5.3 (0.3)	5.0 (0.3)	0.442
Fourth ventricle, mL (VR, %)	3.6 (0.2)	3.7 (0.2)	3.5 (0.2)	0.946
Total SAS, mL (VR, %)	273.1 (17.7)	272.7 (17.4)	273.8 (18.1)	0.651
Convex part of the SAS, mL (VR, %)	72.3 (4.7)	75.3 (4.8)	66.8 (4.4)	0.159
Sylvian fissure and basal cistern, mL (VR, %)	130.2 (8.4)	127.0 (8.1)	136.1 (9.0)	0.350
SAS in the posterior fossa, mL (VR, %)	70.6 (4.6)	70.1 (4.5)	71.3 (4.7)	0.788

^*^Probability value of the Wilcoxon rank-sum test: comparison between pure iNPH and iNPH + AD. iNPH + AD indicates patients with iNPH who had AD as comorbidity.

VR, volume ratio which is calculated as the volume divided by the total intracranial volume.

AC, anterior commissure; AD, Alzheimer’s disease; BVR, brain per ventricle ratio; CSF, cerebrospinal fluid; CVR, convex subarachnoid space to ventricle ratio; iNPH, idiopathic normal pressure hydrocephalus; MRI, magnetic resonance imaging; PC, posterior commissure; SAS, subarachnoid space; SD, standard deviation; VR, volume ratio.

### Changes in morphological indices after shunt implantation

**Figure 2:**
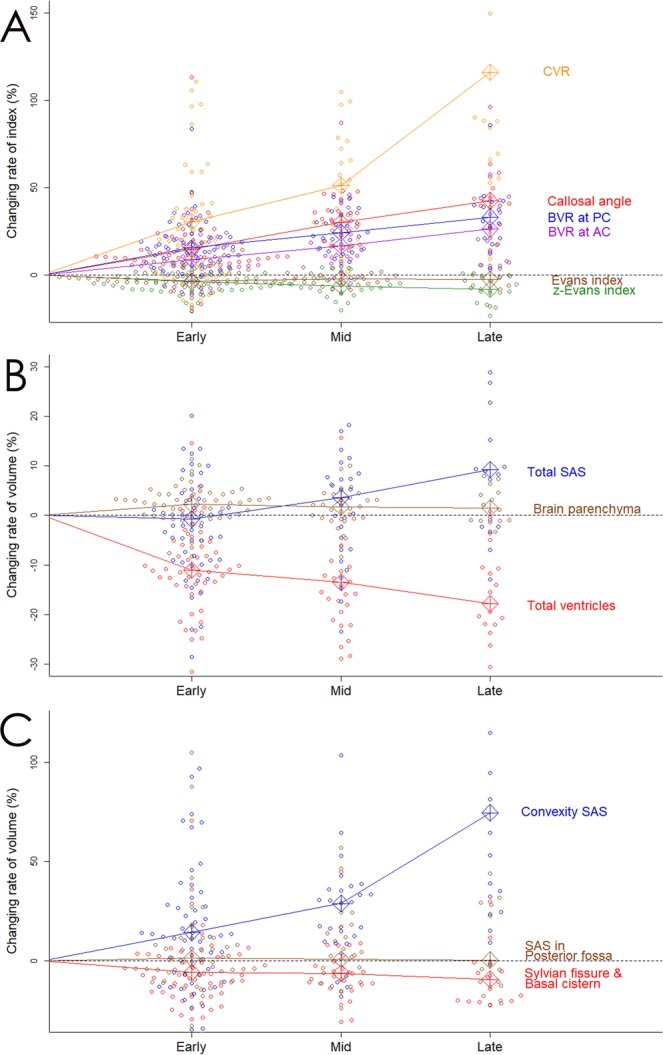
Bee swarm plots of the changing rate of indices and segmented volumes following shunt surgery compared to those at baseline magnetic resonance imaging. Graph A shows the distribution and mean value of the relative changes in the following indices; Evans index (brown), z-Evans index (green), callosal angle (red), brain per ventricle ratio (BVR) at the anterior commissure (AC) level (purple) and BVR at the posterior commissure (PC) level (blue), and convexity subarachnoid space (SAS) per ventricle ratio (CVR; orange). Graph B shows the distribution and mean value of the relative volume changes in the brain parenchyma (brown), total ventricles (red), and total SAS (blue). Graph C shows the distribution and mean value of the relative volume changes in the convexity part of SAS (blue), Sylvian fissure and basal cistern (red), and SAS in the posterior fossa (brown).

The mean value of the Evans index remained unchanged throughout the follow-up period, whereas that of the z-Evans index showed gradual and slight decrease (Table 3 and Fig. 2A). The mean rate of change in the BVR at the anterior commissure (AC) level was more than two-fold than that of the z-Evans index but less than that of the BVR at the PC level. The mean rate of change in the callosal angle was the largest among the two-dimensional (2D) parameters: it was 13% early after VPS, and then increased to > 30% late after VPS.

**Table 3:** Mean values of indices and volumes on MRI before and after shunt surgery.

Characteristics	Pre-shunt (n = 54)	Early (n = 54)	Mid (n = 28)	Late (n = 16)
Evans index (mean ± SD)	0.32 ± 0.06	0.31 ± 0.06	0.33 ± 0.06	0.34 ± 0.05
z-Evans index (mean ± SD)	0.44 ± 0.06	0.42 ± 0.05	0.41 ± 0.05	0.41 ± 0.06
Callosal angle (mean ± SD)	63.5 ± 18.5	72.0 ± 19.6	83.5 ± 24.2	84.5 ± 20.0
BVR at the AC (mean ± SD)	0.72 ± 0.17	0.79 ± 0.16	0.83 ± 0.15	0.84 ± 0.19
BVR at the PC (mean ± SD)	0.89 ± 0.23	1.10 ± 0.25	1.07 ± 0.30	1.09 ± 0.24
CVR (mean ± SD)	0.47 ± 0.20	0.60 ± 0.29	0.66 ± 0.23	0.72 ± 0.31
Brain parenchyma, mL (VR, %)	1107 (71.8)	1131 (73.4)	1116 (73.3)	1099 (73.9)
Total CSF, mL (VR, %)	436.8 (28.2)	412.4 (26.6)	408.7 (26.7)	389.4 (26.1)
Total ventricle, mL (VR, %)	163.7 (10.5)	144.5 (9.3)	141.2 (9.2)	139.4 (9.3)
Bilateral ventricle, mL (VR, %)	155.0 (10.0)	136.3 (8.8)	134.1 (8.8)	135.8 (9.0)
Third ventricle, mL (VR, %)	5.2 (0.3)	4.8 (0.3)	4.3 (0.3)	4.1 (0.3)
Fourth ventricle, mL (VR, %)	3.6 (0.2)	3.3 (0.2)	2.9 (0.2)	2.5 (0.2)
Total SAS, mL (VR, %)	273.1 (17.7)	267.9 (17.3)	267.4 (17.5)	250.0 (16.9)
Convex part of the SAS, mL (VR, %)	72.3 (4.7)	80.2 (5.2)	88.1 (5.7)	92.7 (6.2)
Sylvian fissure and basal cistern, mL (VR, %)	130.2 (8.4)	120.7 (7.8)	116.1 (7.6)	100.1 (6.8)
SAS in the posterior fossa, mL (VR, %)	70.6 (4.6)	68.9 (4.4)	63.5 (4.1)	56.5 (3.8)

^*^Values with significant changes from the pre-shunt values by the Wilcoxon signed-rank test.

AC, anterior commissure; BVR, brain per ventricle ratio; CSF, cerebrospinal fluid; CVR, convex subarachnoid space to ventricle ratio; MRI, magnetic resonance imaging; PC, posterior commissure; SAS, subarachnoid space; SD, standard deviation; VR, volume ratio.

### Volumetric changes

At the early phase after VPS, the mean volume of the total intracranial CSF decreased by 24 ± 39 mL from the baseline due to the decrease both in the total ventricles (19 mL) and the total subarachnoid spaces (5 mL), as shown in Table 3. Conversely, the brain volume slightly increased just as much as intracranial CSF decreased, but subsequently there was no further increase in the brain volume throughout the follow-up period (Table 3 and Fig. 2B). At the mid phase after VPS, the mean volumes of the total ventricles and the total subarachnoid spaces were almost unchanged. At the late phase, however, the mean rate of change in the total ventricular volume decreased < 20% again, whereas that of the total subarachnoid spaces increased < 10%. The mean volume of the Sylvian fissure and basal cistern gradually and slightly decreased from 130 mL to 100 mL at the late phase after VPS (Table 3 and Fig. 2C). In contrast, the mean volume of the convexity subarachnoid space considerably increased > 60% late after VPS. The changing rate of the convexity subarachnoid space per ventricle ratio (CVR), which was calculated as the volume of the convexity subarachnoid space divided by the total ventricular volume, was greater than that of any 2D index (Fig. 2A).

### Relationship with clinical outcome

**Figure 3:**
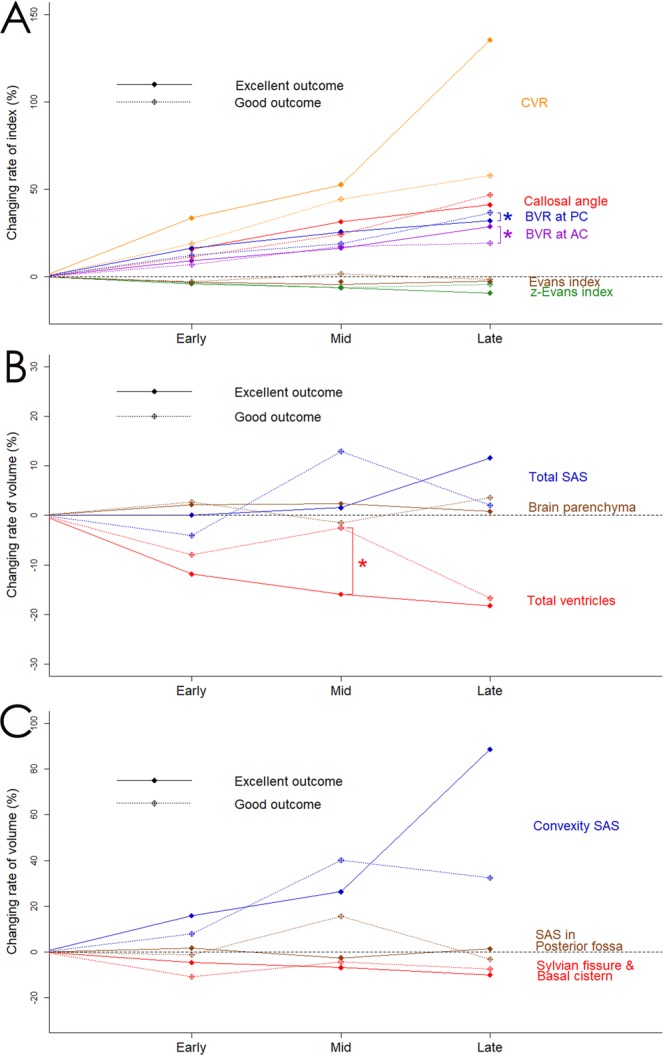
Comparison of indices and segmented volumes after shunt surgery between patients with excellent outcome and those with good outcome. Asterisk (*) indicates statistically significant difference between the patients with excellent outcome (solid line) and those with good outcome (dotted line). Graph A shows the mean value of the relative changes in the following indices: Evans index (brown), z-Evans index (green), callosal angle (red), brain per ventricle ratio (BVR) at the anterior commissure (AC) level (purple) and BVR at the posterior commissure (PC) level (blue), and convexity subarachnoid space (SAS) per ventricle ratio (CVR; orange). Graph B show the mean value of the relative volume changes in the brain parenchyma (brown), total ventricles (red), and total subarachnoid space (SAS; blue). Graph C shows the mean value of the relative volume changes in the convexity part of SAS (blue), Sylvian fissure and basal cistern (red), and SAS in the posterior fossa (brown).

**Figure 4:**
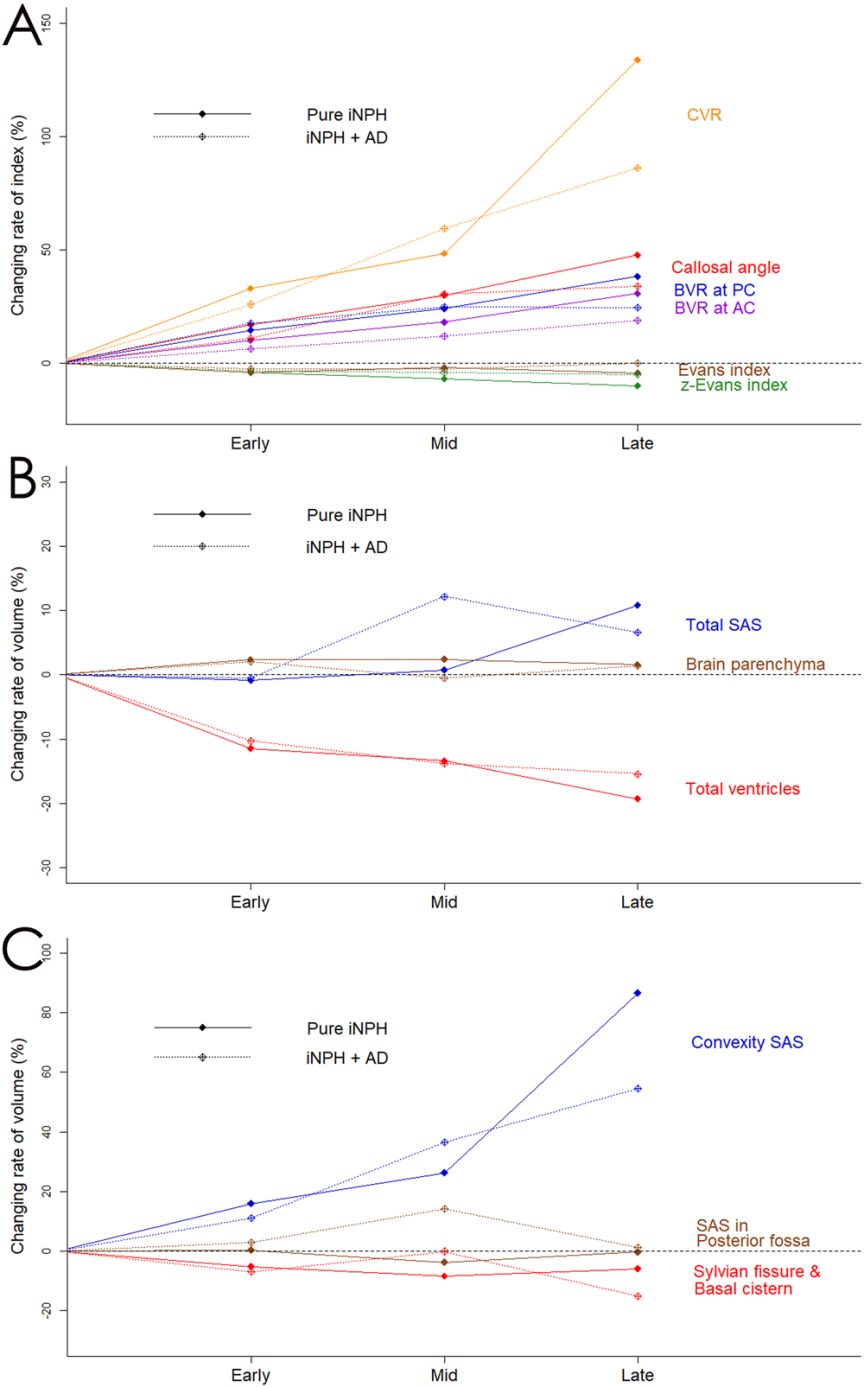
Comparison of indices and segmented volumes after shunt surgery between patients with pure idiopathic normal pressure hydrocephalus (iNPH) and those with co-occurrence of iNPH and Alzheimer’s disease (AD) (iNPH + AD). Filled diamonds and solid lines indicate the patients with pure iNPH and unfilled diamonds and dotted lines indicate those with iNPH + AD. Graph A shows the mean value of the relative changes in the following indices; Evans index (brown), z-Evans index (green), callosal angle (red), brain per ventricle ratio (BVR) at the anterior commissure (AC) level (purple) and BVR at the posterior commissure (PC) level (blue), and convexity subarachnoid space (SAS) per ventricle ratio (CVR; orange). Graph B shows the mean value of the relative volume changes in the brain parenchyma (brown), total ventricles (red), and total subarachnoid space (SAS; blue). Graph C shows the mean value of the relative volume changes in the convexity part of SAS (blue), Sylvian fissure and basal cistern (red), and SAS in the posterior fossa (brown).

Among the iNPH-specific indices, the BVRs at the AC and PC levels late after VPS had a statistically significant difference between excellent and good outcomes (Fig. 3A). The mean volume of the total ventricles in the patients with excellent outcome continued gradually decreasing throughout the follow-up period, whereas that in the patients with good outcome conversely increased at the mid phase after VPS (Fig. 3B). However, the mean volume of the convexity subarachnoid space in the patients with excellent outcome gradually increased until late after VPS, whereas that in the patients with good outcome had a small increase (Fig. 3C). Although the patients with pure iNPH tended to have higher changing rates of the morphological indices and segmented volumes than those with co-occurrence of iNPH and AD, there was no statistical difference (Fig. 4).

## Discussion

**Figure 5:**
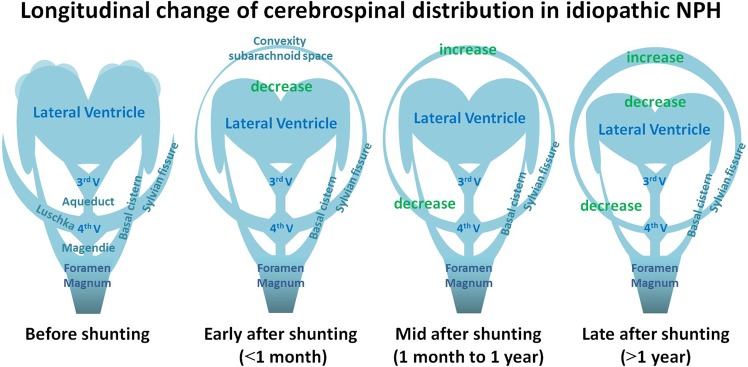
Schematic diagram showing the longitudinal change of CSF in patients with iNPH. The iNPH-specific CSF distribution named disproportionately enlarged subarachnoid space hydrocephalus (DESH) before shunting (left) reverted to a normal distribution in the following three phases; early, mid, and late after ventriculoperitoneal shunt surgery.

Our results indicated that the brain of patients with iNPH expanded <30 mL, which was <2% of the total brain volume, due to a decrease in the intracranial CSF volume just after shunt surgery, but subsequently remained unchanged throughout the follow-up period regardless of improved symptoms gradually. In addition, the disproportionate CSF distribution specific to iNPH reverted to a normal distribution in three phases after shunting as shown in Fig. 5. Early after shunting, both the total ventricles and total subarachnoid spaces had reduced volumes, whereas the convexity part of the subarachnoid space had increased volume. Subsequently, mid after shunting, both the total ventricles and total subarachnoid spaces showed unchanged volumes despite continuous redistribution of the intracranial CSF from the Sylvian fissure and basal cistern to the convexity subarachnoid space. Late after shunting, the total ventricles had reduced volumes again, but the total subarachnoid spaces had conversely increased volumes. However, the callosal angle and BVRs at the AC and PC levels showed gradual increase throughout the follow-up period, independent of the staged changes in the CSF distribution. These findings indicated that the CSF shunt surgery in patients with iNPH causes the convexity part of the brain to move from top to bottom and to regain normal shape via redistribution of intracranial CSF from the ventricles and Sylvian fissure to the convexity subarachnoid space rather than via brain expansion (i.e., like a sponge). We also demonstrated that the Evans index remained absolutely unchanged throughout the follow-up period. These results are in line with those of previous studies^18–21,28,29^. Some reports indicated that the Evans index was not appropriate for evaluating the ventricular size in elderly individuals, especially under the diagnosis of iNPH^7,8,30–32^. Therefore, these findings lead us to concluded that the BVRs at the AC and PC levels or z-Evans index which indicate the z-axial expansion of the bilateral ventricles were appropriate for evaluating ventricular dilatation and shunt effectiveness in patients with iNPH, rather than the Evans index. Additionally, this study revealed that the morphological recovery from brain deformation after shunting was greater posteriorly than anteriorly. Among the 2D indices, the greatest changing rate was that of the callosal angle, followed by was the BVR at the PC level. Recently, Virhammar *et al*. reported that the callosal angle significantly increased 3 months after shunting^21^. In this study, the increase in the both BVRs at the AC and PC levels more than 1 year after shunting was significantly associated with the excellent outcome, although the changing rate in the BVR at the PC level was larger than that of the BVR at the AC level. These findings help to clarify the process of recovery from the symptoms and disproportionate CSF distribution in patients with iNPH after CSF shunt surgery. Additionally, the time-course of the redistribution of the intracranial CSF after shunting may contribute to the next approach to adjust programmable valve pressure setting at the proper timing. For example, the total ventricles in the patients with excellent outcome continued slightly decreasing throughout the follow-up period, whereas those in the patients with good outcome conversely increased at the mid phase after shunting. Even if the shunt valve pressure was appropriate early after shunting, it was actually underdrainage at the mid phase. Because increased ventricular volume after shunting may affect the outcome, we suggest physicians try to lower the shunt valve pressure mid after shunting, if iNPH-related symptoms persist with poor changes in the volume of total ventricle and BVRs at the AC and PC levels.

Our study has some limitations. First, there was a wide range of SDs for the mean values of assessment measures, because of varied size of the ventricles and subarachnoid spaces in patients with iNPH. Therefore, the changes in the indices and segmented CSF volumes have a possibility of large uncertainty. Second, the influence of MRI artefacts due to the shunt valve was small but could not be ignored; nevertheless, we measured the intracranial CSF volume after VPS in patients with shunt valve placement under the occipital scalp. Third, a comorbidity of AD was not confirmed pathologically by brain biopsy, CSF biomarkers or amyloid imaging. Additional information could increase the diagnostic accuracy of AD comorbidity^26,27,33^, although CSF biomarkers at the spinal tap test in iNPH were fluctuate and it is difficult to determine clear cutoff values of them between pure iNPH and iNPH + AD^34^. Finally, the participants in this study did not undergo MRI at the scheduled time-point. There is a possibility of the selection bias that the timing of follow-up MRI was not fixed. Therefore, several novel findings in this study should be further assessed in a prospective cohort study with scheduled timing of follow-up MRI.

In conclusion, the brain parenchyma expanded only 2% from the baseline brain volume early after shunting and remained subsequently unchanged, although CSF distribution considerably changed throughout the follow-up period. Changes in CSF distribution occurred in three phases after shunt implantation. The ventricular volume decreased at the early and late phases, whereas the volume of the total subarachnoid spaces slightly decreased at the early phase, conversely increasing at the late phase. Especially, the volume of the convexity subarachnoid space markedly increased throughout the follow-up period. Both decreased ventricular volume and increased convexity subarachnoid space volume were important for evaluating shunt effectiveness. Therefore, we recommend CVR, which can measure directly the volumetric change both in the ventricles and convexity subarachnoid space, as a 3D index. However, a 3D index is difficult to measure in clinical routine. As simple 2D indices, the callosal angle, BVR at the PC level, BVR at the AC level, and z-Evans index were greatly changed after shunt surgery (in that order), whereas the Evans index remained unchanged throughout the follow-up period. Among them, we recommend the BVRs at the AC and PC levels, because they were significantly associated with better clinical outcomes or comorbid AD. These novel findings may contribute to future studies of the pathogenesis of iNPH and to developing a future treatment approach based on the specific CSF distribution in patients with iNPH.

## Methods

### Ethical approval and patient consent

The study design and protocol were approved by the ethics committee for human research at Rakuwakai Otowa Hospital (IRB Number: Rakuoto-Rin-14-003). After the patients or their relatives provided written informed consent, their private information was anonymized in a linkable manner. The methods were performed in accordance with the approved guidelines outlined in the Declaration of Helsinki.

### Study population

Details of the clinical data collection, image acquisition, and segmentation and quantification of the ventricles and subarachnoid spaces are described in our previous publications^7,8,16^. Volumetric data of T2-weighted 3D-SPACE sequence were prospectively collected from November 2013 to June 2018 on 3-Tesla MRI scanner (MAGNETOM Skyra; Siemens AG, Muenchen, Germany) before and after VPS surgery in consecutive 72 patients diagnosed with iNPH. Three patients who underwent VPS via frontal approach were excluded, because the artefacts from the metal parts in the shunt valve affected the volumetric analysis on MRI. Three patients who underwent lumboperitoneal shunt surgery were excluded, because the different locations of CSF drainage might influence changes in CSF distribution after shunt surgery. Two patients who were diagnosed with shunt malfunction and required shunt revision about 1 year after the first shunt surgery were excluded, because we cannot judge when the shunt was obstructed, or how long it was effective. Additionally, 10 patients who underwent the first follow-up MRI > 1 month after VPS were excluded. Finally, 54 patients with iNPH who underwent the first follow-up MRI within 1 month after VPS via parieto-occipital approach were included in this study. Among them, 19 patients with iNPH (35%) had a comorbidity of AD, based on the comprehensive assessment of their symptoms, prescription of cholinesterase inhibitors, and findings on MRI and single-photon emission computed tomography, according to the current recommendation from the National Institute on Aging–Alzheimer’s Association workgroups^35^.

### Ventriculo-peritoneal shunt and valve pressure adjustment

Details of the surgical procedure for VPS via parieto-occipital approach have been described in previously^36^. Briefly, to insert the ventricular catheter precisely, we routinely conducted preoperative virtual 3D simulation by using the SYNAPSE 3D workstation (Fujifilm Medical Systems, Tokyo, Japan). A shunt valve system including a Codman Hakim programmable valve with a Siphon-Guard^®^ (Integra LifeSciences Corporation, Plainsboro, NJ, USA) was used in 12 patients until March 2016; after that date, 42 patients underwent VPS by using an MRI-resistant Codman CERTAS^®^ Plus programmable valve with a Siphon-Guard^®^ (Integra LifeSciences Corporation, Plainsboro, NJ, USA). The mean values of age, several indices, and volumes were not significantly different between the two groups. The opening pressure of the shunt valve was set based on the patient’s height and weight per Miyake’s quick reference table^37^. All patients were evaluated for over- or underdrainage of CSF based on the changes in the symptoms and CT scan within 10 days after shunt implantation, and subsequently, every 1 month up to 6 months and every 3 months after 6 months. The valve pressure was readjusted in stepwise setting for the Codman CERTAS Plus valve or by intervals of 2 cmH_2_O for the Codman Hakim programmable valve as required. Concretely, in case of insufficient improvement of patients’ symptoms, defined as CSF underdrainage, the valve pressure was lowered; in case of orthostatic headache or subdural effusion on CT scan, defined as CSF overdrainage, the valve pressure was raised. If the valve pressure was readjusted, we checked the patients’ conditions and subdural effusion through CT scan within 1 month after readjustment. In this study, the follow-up MRI finding of apparent subdural effusion or hematoma was absent, and no patient required additional surgery for subdural hematoma during the follow-up period. Clinical outcomes were classified into excellent, good, and unsatisfactory^36^. An excellent outcome was defined as the regained ability to perform outdoor activities that was grade 0 to 2 on the modified Rankin Scale and a ≥ 2-point improvement on the Japanese iNPH grading scale. A good outcome was defined as a 1-point improvement on the modified Rankin Scale or iNPH grading scale but needing some support to perform outdoor activities, which was graded 3 to 4 on the modified Rankin Scale. An unsatisfactory outcome was defined as unchanged or worsening symptoms and persistent severe disability that was grade 5 on the modified Rankin Scale. Table 4 shows the changes in modified Rankin Scale, iNPH grading scale, times on the 3-m timed up-and-go test and 10-m straight walk test and scores of mini-mental state examination and frontal assessment battery at the baseline MRI and the first follow-up MRI. The patients with excellent outcome had significantly mild grade on the baseline modified Rankin Scale and significant improvement on modified Rankin Scale at the first follow-up MRI, compared with those with good outcome. Additionally, the patients with excellent outcome significantly improved the total and gait scores of iNPH grading scale at the first follow-up MRI, compared with the patients with good outcome. However, the mean values and changes of quantitative measurements did not have significant differences between the excellent and good outcomes. The mean time on 3-m timed up-and-go test was shortened by >10 seconds at the first follow-up MRI in both groups of the excellent and good outcomes.

**Table 4:** Change in clinical scores at the baseline and the first follow-up MRI in the groups of excellent outcome and good outcome.

	Excellent (n = 43)		Good (n = 11)		*P* value*
	At baseline	At 1^st^ MRI	At baseline	At 1^st^ MRI	
mRS	2.57 ± 0.67	1.84 ± 0.69	2.82 ± 0.98	2.55 ± 0.82	**0.012**
∆mRS		0.72 ± 0.55		0.27 ± 0.47	**0.026**
iNPHGS total	6.36 ± 1.96	4.28 ± 1.92	6.36 ± 2.50	5.45 ± 2.81	0.118
∆total		2.07 ± 1.47		0.91 ± 1.45	**0.007**
gait	2.52 ± 0.51	1.56 ± 0.59	2.45 ± 0.69	2.09 ± 0.94	**0.024**
∆gait		0.98 ± 0.46		0.36 ± 0.67	**<0.001**
cognitive	1.83 ± 0.88	1.44 ± 0.80	2.00 ± 0.89	1.73 ± 0.90	0.255
∆cognitive		0.37 ± 0.62		0.27 ± 0.47	0.743
urinary	2.00 ± 0.96	1.28 ± 0.85	1.91 ± 1.30	1.64 ± 1.21	0.387
∆urinary		0.72 ± 0.77		0.27 ± 0.47	0.060
TUG	26.2 ± 30.4	13.5 ± 10.0	32.9 ± 33.7	16.7 ± 7.67	0.058
∆TUG		12.7 ± 29.4		16.4 ± 27.0	0.761
10 M Walk	18.4 ± 17.3	10.2 ± 15.5	19.7 ± 20.9	12.2 ± 6.72	0.195
∆10 M Walk		8.2 ± 15.8		7.6 ± 21.3	0.232
MMSE	21.6 ± 5.6	23.6 ± 5.0	21.5 ± 6.7	23.1 ± 5.1	0.777
∆MMSE		1.7 ± 4.5		2.2 ± 2.6	0.986
FAB	9.58 ± 2.9	11.4 ± 3.1	10.0 ± 4.1	10.5 ± 3.5	0.573
∆FAB		1.7 ± 2.8		0.7 ± 3.2	0.240

Data are presented as the mean ± standard deviation unless otherwise noted.

∆ indicates the difference between score at the baseline and that at the first follow-up MRI.

^*^Probability value of the Wilcoxon rank-sum test: comparison between excellent and good outcomes

mRS, modified Rankin Scale; iNPHGS, idiopathic normal pressure hydrocephalus grading scale; TUG, timed 3-m up-and-go test; 10 M Walk, 10-m straight walking test; MMSE, mini-mental state examination; FAB, frontal assessment battery.

### Image acquisition and volumetric analysis

**Figure 6:**
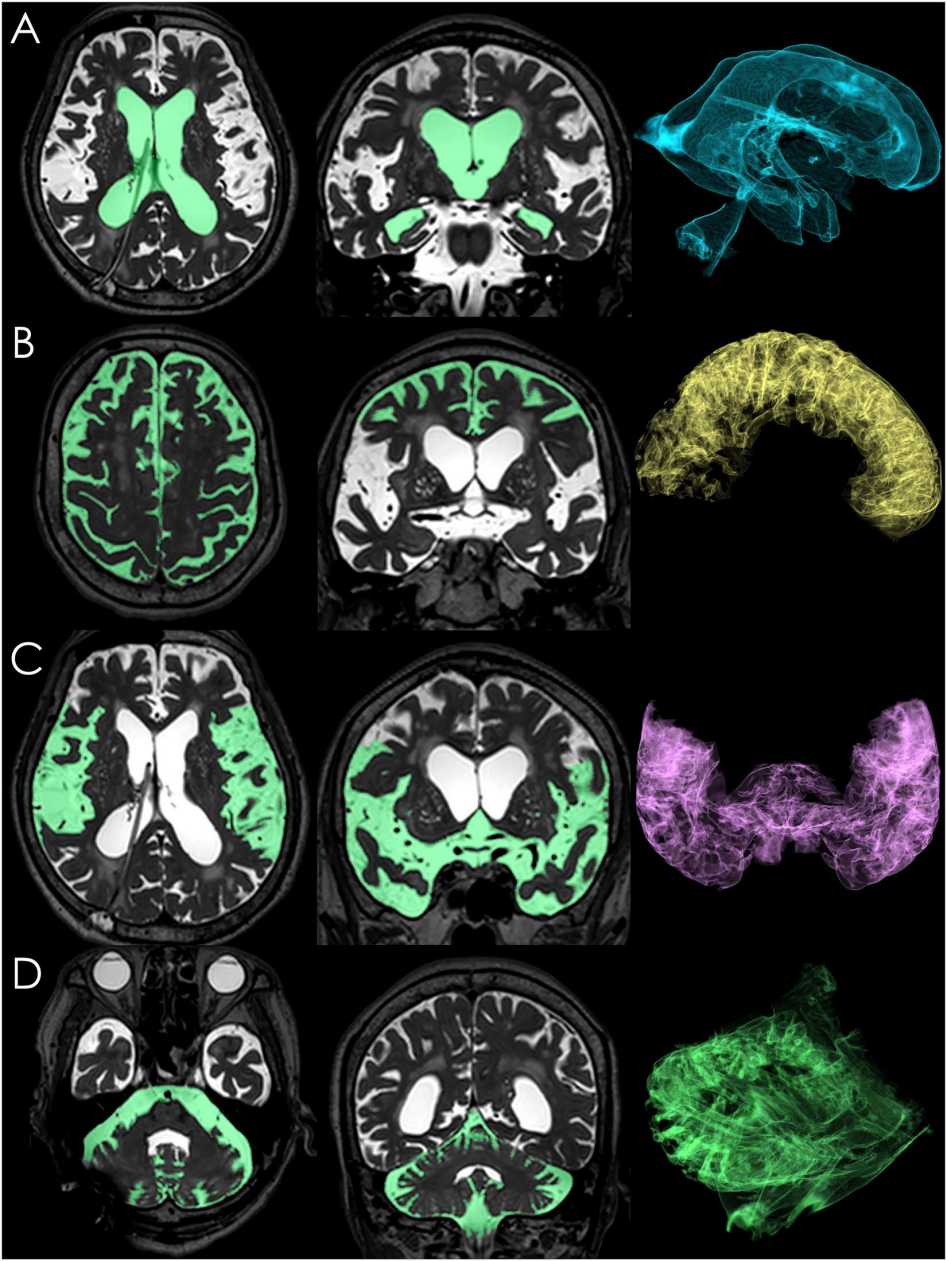
Segmentation of the total ventricles and three parts of the subarachnoid spaces. These images were created on the SYNAPSE 3D workstation (Fujifilm Medical Systems, Tokyo, Japan) from a three-dimensional (3D) T2-weighted 3-Tesla sequence 10 days after ventriculoperitoneal shunting via parieto-occipital approach in a patient diagnosed with co-occurrence of idiopathic normal pressure hydrocephalus (iNPH) and Alzheimer’s disease (AD). The ventricles were manually extracted from intracranial cerebrospinal fluid (CSF) space by enclosing in a free shape. (**A**) The total subarachnoid spaces were segmented by subtraction of the total ventricles from total intracranial CSF spaces. The convexity subarachnoid space (**B**) was manually segmented from the total subarachnoid spaces per the anatomical landmarks of the basal interhemispheric cistern, Sylvian fissure, and tentorium cerebelli. From the residual subarachnoid spaces, the subarachnoid space in the posterior fossa (**D**) was segmented with reference to the anatomical landmarks of the tentorium cerebelli, chiasma and optic tract. There was a small MRI artefact in the right occipital posterior fossa. The Sylvian fissure and basal cistern (**C**) were segmented by subtraction of the convexity subarachnoid space and subarachnoid space in the posterior fossa from the total subarachnoid spaces. The convexity subarachnoid space per ventricle ratio (CVR), which was defined as the volume of the convexity subarachnoid space (178 mL) divided by the total ventricular volume (118 mL), was 1.51.

Because all patients had shunt valve placement under the right occipital scalp, MRI artefacts due to the shunt valve system on the T2-weighted 3D sequence slightly affected the CSF volume of the subarachnoid space in the posterior fossa (Fig. 6). The volumetric data were entered on a 3D workstation, where the intracranial space was semi-automatically segmented with recent technologies combined the user-steered live-wire segmentation, edge-guided nonlinear interpolation, and automatic extraction of continuous objects. After that, the intracranial space was subdivided into the brain parenchyma and intracranial CSF space through a simple threshold algorithm. Finally, the intracranial CSF spaces were manually segmented into the total ventricles, convexity subarachnoid space, Sylvian fissure and basal cistern, and subarachnoid space in the posterior fossa (Fig. 6 and Video). Each segmented volume was automatically measured by counting the number of voxels. Volume ratios, which were calculated as the measured volumes divided by the intracranial volume, were analyzed to eliminate the effect of head size.

### Morphological indices specific to NPH

**Figure 7:**
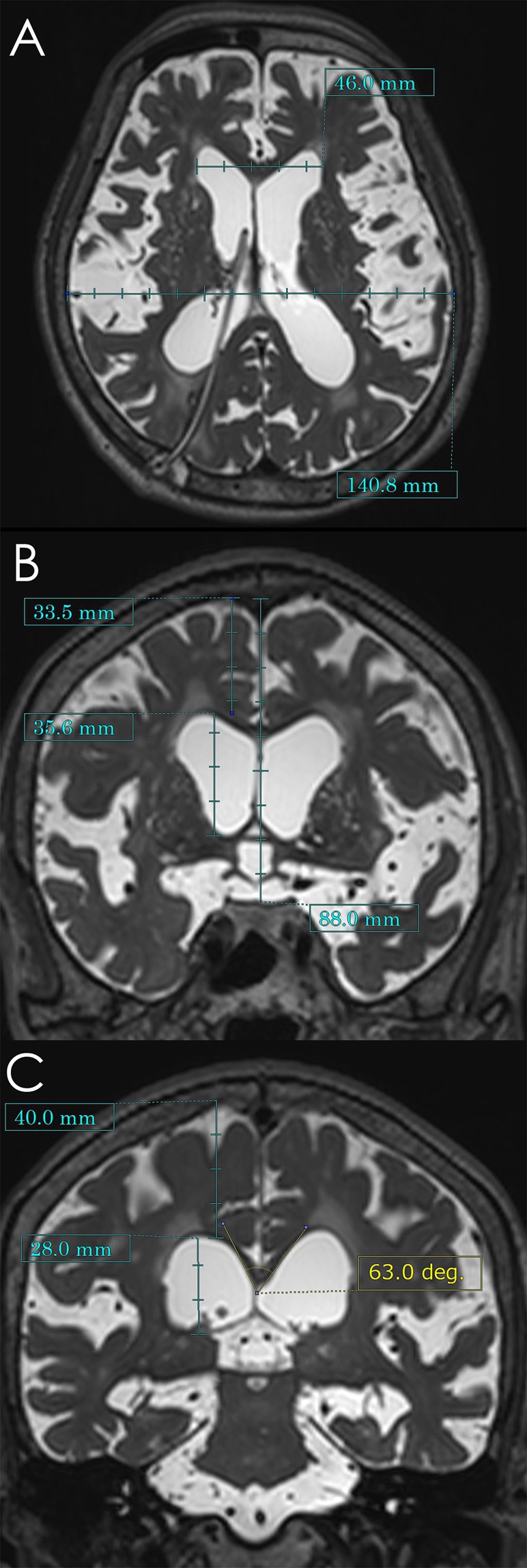
Morphological indices specific to idiopathic normal pressure hydrocephalus (iNPH). These magnetic resonance images are shown for the same patient as Fig. 6. The Evans index was calculated as 0.327 (**A**); the maximum length of the frontal horns of the lateral ventricles (46.0 mm) divided by the maximum cranial length on the same axial plane (140.8 mm). The z-Evans index, which was calculated as the maximum z-axial length of the frontal horns of the lateral ventricles (35.6 mm) divided by the maximum cranial z-axial length at the midline (88.0 mm) on the coronal plane just on the anterior commissure (AC), was 0.405 (**B**). The callosal angle was calculated as 63 degrees (**C**) at the roof of the bilateral ventricles on the coronal plane just on the posterior commissure (PC). The brain per ventricle ratios (BVRs) at the AC and PC levels were calculated as 0.941 (**B**) and 1.429 (**C**), respectively.

Figure 7 shows the measured indices early after VPS in the same patient with co-occurrence of iNPH and AD as Fig. 6. The Evans index (Fig. 7A) was measured as the maximal width of the frontal horns of the lateral ventricles to the maximal width of the internal diameter of the cranium based on the x-dimension^38^. The z-Evans index (Fig. 7B) was measured as the maximum z-axial length of the frontal horns of the lateral ventricles to the maximum cranial z-axial length on the coronal plane, which was perpendicular to the anteroposterior commissure plane on the AC^7^. The callosal angle (Fig. 7C) was measured as the angle of the roof of the bilateral ventricles on the coronal plane at the PC level^5^. The BVRs at the AC and PC levels (Fig. 7B,C) were calculated as the maximum width of the brain just above the lateral ventricles divided by the maximum width of the lateral ventricles on the reference coronal planes at the AC and PC levels, respectively^8^. The CVR was defined as the volume of the convexity-subarachnoid space divided by the total ventricular volume^8^.

Absolute and relative changes in the morphological indices and volumes in brain parenchyma, total ventricles, and three parts of subarachnoid spaces after VPS were compared to those before VPS. Absolute changes were calculated as the baseline indices and volumes minus those after VPS, and relative changes were calculated as the absolute changes divided by the baseline indices or volumes × 100 (%).

### Statistical analysis

Absolute and relative changes in mean values and standard deviations (SDs) for the segmented volumes and indices after VPS were compared using Wilcoxon signed-rank test. The frequency of follow-up MRI examinations and the period from VPS to MRI varied widely: 54 patients underwent the first follow-up MRI from 5 to 29 days (median: 11 days) after VPS, 32 patients underwent the second MRI from 36 to 742 days (median: 178 days), 12 patients underwent the third MRI from 366 to 1270 days (median: 559 days). Therefore, we categorized the period from VPS to follow-up MRI into <1 month (early, 54 patients), 1 month to 1 year (mid, 28 patients), and >1 year (late, 16 patients). The Wilcoxon rank-sum test was used to compare the mean values between patients with and without AD. Fisher’s exact test was used to compare the proportions of the 2 groups. Statistical significance was assumed at a probability (*P*) value of less than 0.05. All missing data were treated as deficit data that did not affect other variables. Statistical analysis was performed using R software (version 3.3.2; R Foundation for Statistical Computing, Vienna, Austria; http://www.R-project.org).

## Supplementary information

Video
